# Ultrastable low-cost colloidal quantum dot microlasers of operative temperature up to 450 K

**DOI:** 10.1038/s41377-021-00508-7

**Published:** 2021-03-18

**Authors:** Hao Chang, Yichi Zhong, Hongxing Dong, Zhenyu Wang, Wei Xie, Anlian Pan, Long Zhang

**Affiliations:** 1 Key Laboratory of Materials for High-Power Laser, Shanghai Institute of Optics and Fine Mechanics, Chinese Academy of Sciences, 201800 Shanghai, China; 2 State Key Laboratory of Precision Spectroscopy, School of Physics and Electronic Science, East China Normal University, 200241 Shanghai, China; 3 Hangzhou Institute for Advanced Study, University of Chinese Academy of Sciences, 310024 Hangzhou, China; 4 State Key Laboratory of High Field Laser Physics, CAS Center for Excellence in Ultra-intense Laser Science, Shanghai Institute of Optics and Fine Mechanics, Chinese Academy of Sciences, 201800 Shanghai, China; 5 Center of Materials Science and Optoelectronics Engineering, University of Chinese Academy of Sciences, 100049 Beijing, China; 6 Key Laboratory for Micro-Nano Physics and Technology of Hunan Province, State Key Laboratory of Chemo/Biosensing and Chemometrics, College of Materials Science and Engineering, Hunan University, 410082 Changsha, China

**Keywords:** Micro-optics, Quantum dots, Semiconductor lasers

## Abstract

Quantum dot microlasers, as multifunctional optical source components, are of great importance for full-color high-pixel display, miniaturized coherent lighting, and on-chip integrated photonic and electronic circuits. Since the first synthesis of colloidal quantum dots (CQD) in the 1990s, motivation to realize high-performance low-cost CQD micro-/nanolasers has been a driving force for more than three decades. However, the low packing density, inefficient coupling of CQDs with optical cavities, and the poor thermal stability of miniaturized complex systems make it challenging to achieve practical CQD micro-/nanolasers, especially to combine the continuous working ability at high temperatures and the low-cost potential with mass-produced synthesis technologies. Herein, we developed close-packed CQD-assembled microspheres and embedded them in a silica matrix through the rapid self-aggregation and solidification of CdSe/ZnS CQD. This technology addresses the core issues of photoluminescence (PL) quenching effect and low optical gain in traditional CQD laser research. High-efficiency low-threshold CQD microlasers are demonstrated together with long-playing (40 min) working stability even at 450 K under pulsed laser excitation, which is the highest operational temperature for CQD lasers. Moreover, single-mode CQD microlasers are obtained with tunable wavelengths across the entire visible spectral range. The chemosynthesis process supports the mass-produced potential of high-density integrated CQD microlasers, promoting CQD-based low-cost high-temperature microdevices.

## Abstract (web summary)

The three core problems of CQD laser research and the corresponding solutions for achieving high-temperature CQD lasers.

## Graphical abstract

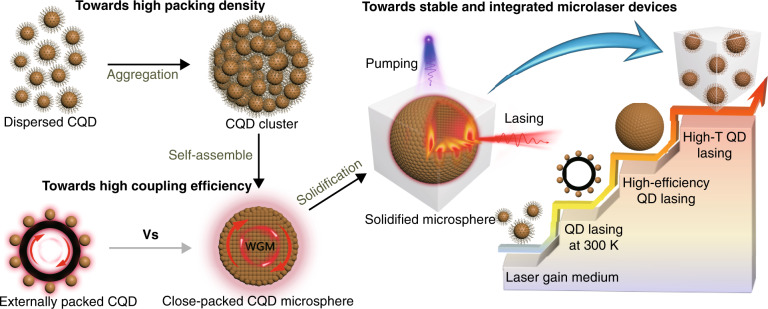

## Introduction

Low-dimensional CQD have attracted significant attention because of their unique structures, extraordinary optical properties, and low-cost preparation processes^1–11^. The well-separated delta function-like density of electronic states and their large optical oscillator strength promise CQD with low threshold and temperature-insensitive optical gain that make them excellent candidates for laser gain materials^12,13^. In the past few decades, considerable efforts have been devoted to investigating and developing CQD lasers, and significant progress has been achieved. Generally, CQD lasers could be obtained by inserting the solidified monodisperse CQD layer into the etching structures^14^, including distributed feedback gratings^15–18^ and photonic crystals^19^. Another typical method, the most direct one, is coating highly concentrated CQD solutions on microcavities^20,21^ or forming CQD film^22–26^ to obtain lasers. In fact, the research of CQD microlasers is still in the initial stage, focusing on how to obtain high lasing performance, such as high-quality factors, narrow line widths, and low thresholds. Even so, the present generation of CQD lasers still faces many difficulties. The core problems are the relatively low gain packing density and inefficient coupling of CQD with optical cavities, which significantly challenge the improvement of basic lasing performance. Moreover, with the development trend of miniaturization and integration for the next generation of optoelectronic devices^27,28^, the heat dissipation problem cannot be effectively solved by the traditional water-cooling method in highly integrated microdevices, which requires the miniaturized optical components to work stably at high temperatures. However, poor stability, especially the PL quenching effect at high temperatures, is recognized as another critical issue limiting CQD for further practical applications^29–31^. Although solidified monodisperse CQD in the protective matrix can eliminate the surface defect states and improve the temperature tolerance of CQD^32,33^, they still suffer from low gain density and inefficient coupling in current CQD laser research.

To address these issues, we developed a novel assembly technique combined with the sol–gel method to fabricate CdSe/ZnS CQD-assembled microspheres (CQDAMs) solidified in a silica matrix, which not only guarantees that the CQDAMs work stably at high temperatures but also solves the problems of gain packing density and coupling efficiency. We first achieved single-mode lasing based on solidified CQDAMs with operative temperatures up to 450 K. So far, this is the highest operational temperature for CQD microlasers. Even if they continuously work in such a high-temperature environment, the stable output of lasing pulses can be maintained for 40 min. By changing the composition and/or size of CQD, single-mode lasing can be extended to the entire visible spectral range. Moreover, the solution-processable method has the advantages of low cost and potential for mass production. It does not require complex optical cavity processing, which means no expensive equipment or extremely complex processing is required. Meanwhile, these CQDAMs lasers can be highly integrated into a micro-substrate, and also applicable to other kinds of semiconductor nanoparticles, which promote predictable commercial application value in high-temperature low-cost micro-integrated optoelectronic devices.

## Results

**Fig. 1:**
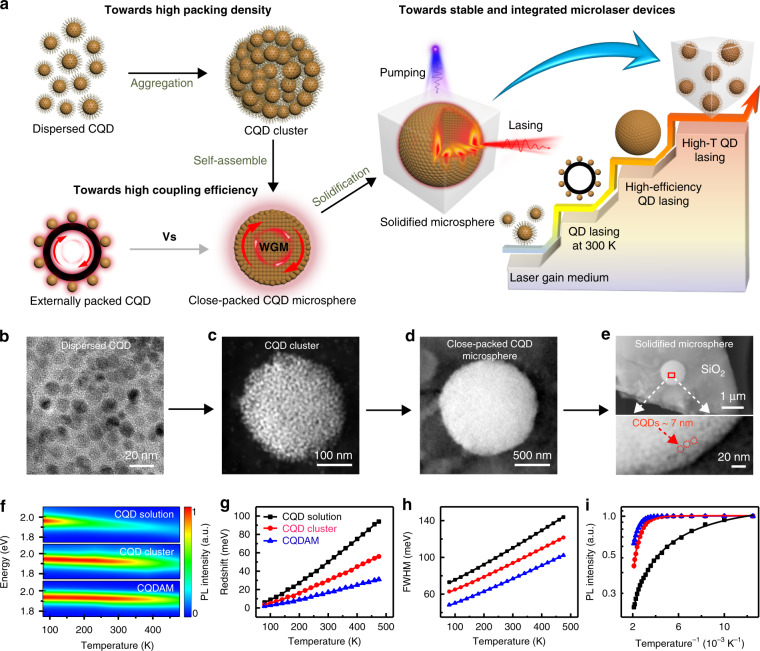
Schematic of experimental design and morphology characteristics of different CQD structures. **a** Schematic of the three core problems of CQD laser research and the corresponding solutions of our work for achieving high-temperature CQD lasers. Dispersed CQD self-assembling into closely packed CQD cluster to achieve high packing density, then to the CQD-assembly microsphere to achieve high coupling efficiency, finally to the solidified microsphere to achieve stable and integrated high-T laser. The CQD-assembly microsphere can serve as both gain medium and microcavity. Lights travel inside the WGM microcavity due to the total internal reflection at the resonator boundary to achieve high coupling efficiency. CQDAMs are solidified in silica matrix through sol–gel method to ensure stable working at high temperatures. **b**–**e** TEM images of different CQD structures, corresponding to the model in (**a**). **f** Contour plots of the temperature-dependent emission from different CQD structures with the temperature varied from 77 to 475 K under the same excitation conditions. **g**–**i** Evolution of emission peak redshifts (**g**), spectral full-width at half-maximum (FWHM) (**h**), and PL intensity (**i**) for the three different CQD structures with the increase of temperature

Figure 1a shows a schematic of the three core problems of CQD lasers research and our solutions for achieving ultrastable high-temperature CQD lasers. Generally, methods for realizing CQD lasers require an external cavity structure, and this can be roughly divided into two categories. The first is the insertion of solidified monodisperse CQD layers into the etching structures, including distributed feedback gratings and photonic crystals. This type of CQD lasers generally suffers from extremely complex processing techniques, expensive manufacturing costs, and relatively low gain density. The second category involves coating highly concentrated CQD solutions on microcavities or forming CQD films to obtain lasers. However, these usually suffer from the inefficient coupling between CQD and microcavities, leading to low Q factors and high thresholds. Unlike the traditional methods, high-quality solidified CQDAMs were obtained using innovative self-assembly and sol–gel method. First, commercially available dispersed CdSe/ZnS core/shell CQD was used in our experiment (Supplementary Fig. S1). The decreased electron–hole overlap remarkably suppressed the nonradiative Auger recombination rate, making such core/shell CQD very favorable as a gain medium^34,35^. The PL quantum yield of CQDs is up to 80%. The high quantum efficiency suggests that the oscillator strength of excitons in our CQDs is not significantly influenced by the decrease of electron–hole overlap. At least, the suppression effect of Auger recombination is stronger than the decrease of oscillator strength during the decreasing process of electron–hole overlap. Then, we deliberately broke the equilibrium of CQD in the sol process, resulting in rapid self-aggregation of CQD to form close-packed CQD clusters, which addresses the issue of low packing density. Next, slowly evaporating the solution enabled CQD of similar size to aggregate more tightly, thus achieving regular geometrical CQDAMs. The size of CQDAMs can be controlled by varying the concentration of the CQD and the stirring rate in the sol–gel process (Supplementary Fig. S2). This new CQDAM structure is an ideal combination of the gain medium and optical microcavity, thus solving the problems of the low gain density and coupling efficiency of CQD laser research. Finally, the sol was rapidly solidified through a gel process to form a protective matrix. The CQDAMs embedded in the silica matrix obtain high thermal tolerance (Supplementary Figs. S3 and S4), which could solve the problem of poor thermal stability and achieve integrated high-temperature CQD microlasers. Figure 1b–e shows the objective TEM images of different CQD structures corresponding to the model in Fig. 1a. These CQDAMs samples maintain superior stability in the atmospheric environment for over 2 years with little change in optical properties.

The key difficulty to realize the high-temperature CQD lasers is to overcome the PL quenching^29,30^ and the low optical gain problem at high temperature. Our original idea from dispersed CQD to CQDAMs is reasonable and efficient for the optimization of the intrinsic physical parameters of the gain medium to solve the problem. Temperature-dependent PL spectra from the CQD solution, CQD cluster, and CQDAM are shown in Fig. 1f. Interestingly, the optical properties are gradually optimized during the synthesis process from the initial solution of dispersed CQD to the final product of solidified CQDAMs, which confirms the validity of our design ideas and the sample preparation methods (Part II, SI). In principle, the FWHM of the PL peak is significantly influenced by the nonradiative scattering rate between excitons and thermally induced phonons, which decreases the nonradiative lifetime and the radiative efficiency of excitons^36,37^. This is one of the reasons for the quenching of the PL intensity at high temperatures. Meanwhile, the defect states and other nonradiative centers also make notable contributions^38,39^. Thermal-induced surface ligand detachment and atomic dislocation are the origin of nonradiative trapping centers and surface defects of CQD. For solidified CQDAMs, the smallest increase of peak FWHM at high temperature suggests the effective suppression of phonon scatterings. Moreover, additional surface passivation that refers to the further elimination of surface defect states during the CQDs self-assembly process besides the passivation of the silica matrix, and the closely packed microsphere structure significantly decreases the density of inner defects, which efficiently overcome the PL quenching effect and ensure a strong optical gain. The CQDAMs have the smallest redshift energy of the PL peak (Fig. 1g), the smallest increase of peak FWHM (Fig. 1h), and the smallest reduction of PL intensity (Fig. 1i) compared to those of other structures, which provide favorable conditions for achieving a stable laser at high temperature.

**Fig. 2:**
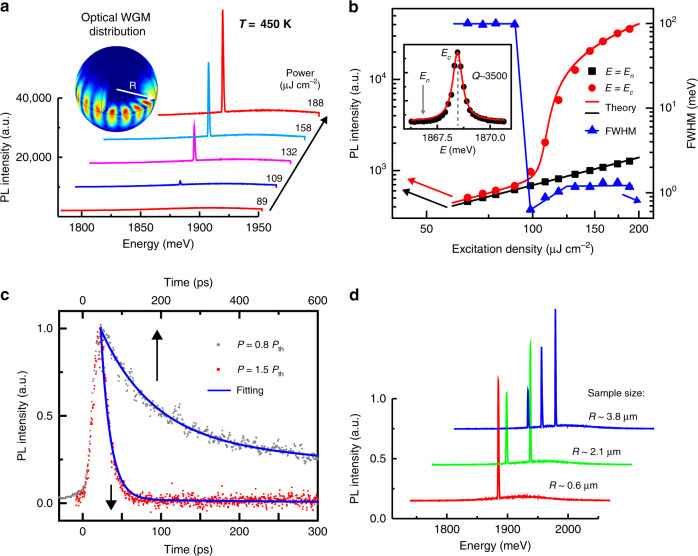
Single-mode lasing characteristics of CQDAMs at high temperature. **a** Power-dependent emission spectra of a typical CQDAM at 450 K. Inset: three-dimensional optical WGM distribution based on a microsphere with radius *R* = 590 nm by theoretical simulation. **b** The excitation density VS the PL intensities at the cavity resonance energy (*E*_c_: 1868 meV) and the nonresonant energy (*E*_n_: 1863 meV). Plots of emission FWHM of PL spectra VS excitation density. Inset: Lorentzian fitting of the lasing peak with an FWHM of ∼0.6 meV, corresponding to a *Q* factor of ∼3500. **c** Typical PL decay profiles of the emission measured under different excitation densities of 0.8 *P*_th_, and 1.5 *P*_th_. The solid lines are the fitting results based on biexponential decay functions. The spontaneous and stimulated radiation lifetimes are 300 and 13 ps, respectively. **d** The resonant optical modes of CQDAMs with different sizes. Multimode lasing can be obtained and the spacing between two adjacent modes decreases with the increase of cavity size

In our experiment, the solidified CQDAM shows excellent laser performance, while the dispersed CQDs and CQD clusters cannot reach the lasing regime even if they are damaged by the high-excitation power. Figure 2a shows the power-dependent lasing spectra of an individual CQDAM at 450 K. Remarkably, it is the highest operational temperature for CQD lasers to date. This result overcomes the thermal management issue of CQD microlasers, representing a significant step toward the exploitation of CQD for miniaturized and integrated coherent light source applications. The typical lasing performance of CQDAMs can be attributed to the nearly perfect naturally formed spherical WGM cavity with high optical gain, as seen in the inset of Fig. 2a. Figure 2b shows the excitation density dependence of the PL intensity at the cavity resonance energy of 1868 meV and the nonresonant energy of 1863 meV, respectively. The abrupt increase in the stimulated emission intensity indicates the achievement of lasing action with a threshold of ∼98.6 μJ cm^−2^. Theoretical simulation matches the experimental data (Theoretical model, “Methods”). The FWHM of the stimulated spectral peak is 0.6 meV, corresponding to the *Q* factor of ∼3500. More discussions on the *Q* factor of the lasing CQDAM sample could be seen in Supplementary Fig. S5. In addition, it can be noticed that the broadening of the FWHM of the lasing peak (0.6–1.3 meV) occurs with increasing of the pumping density, which may induce a variation of the refractive index of microcavity or exhibition additional nonequilibrium effects by excitation-induced heating^40^ (Fig. 2b). The PL lifetime decreases rapidly with the increase of excitation density, which also supports the photo-stimulated amplification behavior of CQDAMs. Moreover, such lasing behavior can also have a lower threshold at room temperature with *Q* values as high as 13,000 (Supplementary Fig. S5). To further confirm the WGM microcavity effect in these CQDAMs, we performed lasing emission measurements for CQDAMs with different diameters (Fig. 2d). The mode space and the energy centers of lasing peaks can be controlled by adjusting the size of the CQDAMs, which match the theoretical prediction of the confined WGMs (Part III, SI). Thus, we demonstrate a new type of CQD single-mode lasers that works at high temperatures.

**Fig. 3:**
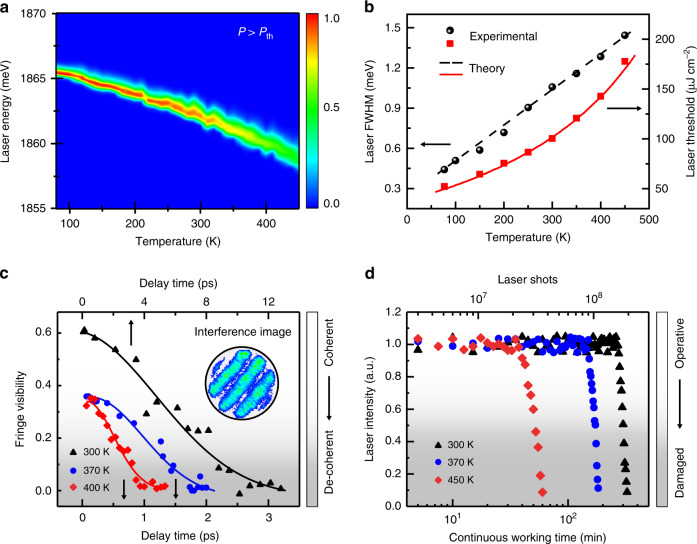
Temperature-dependent laser characteristics of CQDAMs and thermal stability under continuous working conditions. **a** Normalized contour plots of the temperature-dependent lasing spectra from an individual CQDAM at excitation density above the threshold. **b** Temperature-dependent laser FWHM (black circle) and threshold (red square) in the range from 77 to 450 K. **c** The interference fringe visibility of the lasing output by using a Michelson interferometer. The coherent time is obtained by the time-dependent self-interference experiments. The dots are the experimental data, and the lines are the fitting results. The corresponding coherence times at 300, 370, and 400 K are ~5.4, 0.85, and 0.44 ps, respectively. Inset: interference image. **d** Continuous working ability at different temperatures. The stable intense output from an individual CQDAM can be maintained for 40 min with 2.4 × 10^7^ lasing pulses even at 450 K

Figure 3 further elaborates the laser characteristics of CQDAMs at different temperatures. Figure 3a shows contour plots of the temperature-dependent laser spectra of an individual CQDAM with the operation temperature increasing from 77 to 450 K at a fixed excitation density above the threshold. As the increase of temperature, the distortion of the cavity reduces the photon lifetime and increases the FWHM of the lasing peak (Fig. 3b, black dot). The laser threshold rises (Fig. 3b, red block) due to the enhancement of the phonon–exciton scatterings and the reduced cavity photon lifetime. We further discussed the shifting trend of the lasing peak in Fig. 3a (Part II, SI). Meanwhile, the oscillator strength of excitons decreases with the growth of temperature^36,37,41^. These factors accelerate/weaken the rates of non-radiation/radiation recombination, respectively. The theoretical results match the experimental data (Theoretical model, “Methods”). Figure 3c shows the temperature-dependent coherence characters. The typical coherence time of the CQD lasers is ~0.5 ps at 400 K (detailed fitting results in Part III, SI), which is determined by the lifetime of cavity photons. Considering the laser FWHM energy range of 0.6–1.5 meV for different samples at high temperatures, the corresponding range of cavity photon lifetime is about 1.2–0.5 ps, which limits the value of the coherence time. As the temperature decreases, the coherence time increases rapidly. The coherence time could be as long as ~5 ps for high-Q samples working slightly above the lasing threshold at room temperature. Improving the *Q* factor of the cavity and reducing the dephasing of excitons could improve the coherent properties of emission output.

**Fig. 4:**
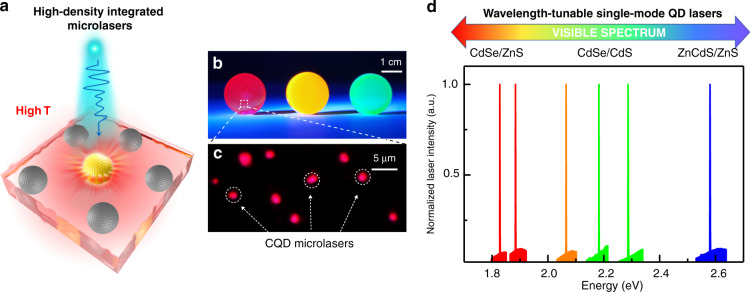
High-density integrated lasers and broad wavelength-tunable single-mode lasers at high temperature based on CQDAMs. **a** Schematic diagram of the mass-produced integrated CQD microlasers working at high temperature. **b** Real-color image of the different CQD-based silica matrix samples excited by ultraviolet light. **c** The corresponding internal enlarged microscopic image under high-excitation condition. The red dots are the lasing CQDAMs at 400 K. **d** Multicolor single-mode lasers coming from CQDAMs of different compositions and/or sizes, the lasing energies of which cover the entire visible range

To analyze the working stability of single-mode lasers, CQDAMs were constantly pumped by a pulsed laser at different temperatures with an excitation density of 1.1 *P*_th_. The real-time-integrated emission intensity varied with the excitation time, as shown in Fig. 3d. Under atmospheric conditions, the lasing output was so robust that it stayed nearly constant for over 270 min over 1.62 × 10^8^ excitation cycles (Fig. 3d, black triangles), which represents more than one order of magnitude longer than that of other CQD lasers^42–44^, indicating the exceptional photostability of these CQDAMs. Even at 450 K, the lasing intensity maintained its initial value for 2.4 × 10^7^ (40 min) sustained pulsed excitation (Fig. 3d, red squares). The long-playing and stable stimulated emission operation observed here indicates that the CQDAM could remain undamaged and robust in long-duration, high-intensity excitation, which shows obvious advantages compared to other colloidal nanocrystals. Such excellent high-temperature lasing performance makes our results novel and useful for progressing towards practical CQD-based lasers with good temperature tolerance. Moreover, the CQDAM lasers could be compatible with high-density integration (Fig. 4a). Figure 4b, c presents an integrated CQD microlasers sample, where multiple CQD microsphere lasers are embedded into a silica matrix with stable outputs at high temperatures. Dozens of CQD microlasers could be integrated into an area of hundreds of square microns, contributing intense light outputs.

Wavelength tunability of microlasers is another pursued goal, which is very important for practical applications^45,46^. Although great efforts have been devoted, a high-temperature broad wavelength-tunable CQD laser has not been achieved yet. The facile microlaser fabrication method we developed can be used as a general paradigm to apply to other aqueous CQD, which allows the lasing wavelength to potentially be tuned across the entire visible spectral range by simply changing the composition and/or size of the CQD. Figure 4d shows the lasing spectra from typical CdSe/ZnS, CdSe/CdS, and ZnCdS/ZnS CQDAMs at high temperatures (>400 K). It can be clearly seen that tunable single-mode lasing is achieved, ranging from 1.82 to 2.67 eV, covering the entire visible spectrum range, which is first reported in CQD systems at high temperatures.

## Discussion

In the research field of nano-/microlaser devices, high-performance low-cost CQDs laser is an important hot topic. Unfortunately, the development is obviously hysteretic considering the coexistence of the multilevel challenges, that is, (1) the basal requirement of excellent lasing performance; (2) the promotional ability to meet the application conditions such as continuous working with high stability, applicability in high-temperature environments; (3) the combination of low-cost production advantage and the merits in previous points (1), (2).

Table 1 and Supplementary Table S1 summarize the main laser parameters for CQD-based micro-/nanolasers reported in previous studies and this work. Remarkably, the lasing performances (lasing threshold, *Q* factor, wavelength tunability, and mode property) of our CQDAMs are excellent. From the point of view of the gain medium, the self-assembled CQDs almost reach the high limit of packing density, ensuring sufficient optical gain. From the point of view of light–matter coupling, such CQDAM samples are used both as gain materials and as optical microcavities, fully improving the light–matter coupling efficiency. From the point of view of optical cavity performance, the spherical WGM microcavity can effectively improve the confinement ability of cavity photons. For a CQDAM sample of volume of about 1 μm^−3^, there could be only a single resonant mode affected in the emission wavelength range. However, the *Q* factor of operative mode could be 10^4^. Most importantly, we combine these three advantages of different aspects together into the CQDAM sample.

**Table 1:** Comparison of main characteristics of reported CQD-based micro/nanolasers

Performance materials	Laser parameters			Commercialization potential			Ref.
	Mode (*Q* factor)	Wavelength tunability	Threshold (μJ cm^−2^)	Tolerable temperature (micro-integrated)	Continuous working laser pulses	Mass-produced potential (hard/easy/great)	
Perovskite CQD	ASE (—)	—	1.2–2800	300 K (✗)	3.2–6 × 10^6^	Spin coating (easy)	^47,48^
	ASE (—)	✓ (1.57–3.12 eV)	12	300 K (✗)	9 × 10^7^	Spin coating (easy)	^24^
	Multimode lasing (—)	—	2000	370 K (✓)	1.2 × 10^6^	Lithography (hard)	^13^
	Multimode lasing (*Q*~920)	✓ (2.1–2.63 eV)	11	300 K (✓)	—	Lithography (hard)	^49^
	Multimode lasing (*Q*~1500)	—	22–910	300 K (✗)	—	Drop casting (easy)	^20,21^
Semiconductor CQD	ASE (—)	—	6–40,000	300 K (✗)	—	Spin coating (easy)	^23,26,39^
	Random lasing (—)	—	4.4–17,100	300 K (✗)	—	Spin coating (easy)	^25,50,51^
	Multimode lasing (*Q*~1300)	—	5.5–60	300 K (✓)	—	Lithography (hard)	^14,15,17,18^
	Single-mode lasing (*Q*~2500)	—	52–6400000	300 K (✓)	—	Lithography (hard)	^16,19^
	Single-mode lasing (*Q*~3500–13,000)	✓ (1.82–2.67 eV)	60–190	450 K (✓)	0.3–1.62 × 10^8^	Chemosynthesis (great)	This work

Besides the above laser parameters, the lasing stability at high temperature is also an important aspect related to commercialization potential. The heat dissipation problem is an intrinsic and inevitable difficulty for the next generation of microchip-integrated lasing devices. In this work, the operative temperature of CQDs microlaser is demonstrated to promote to 450 K. Moreover, the CQDs microlaser can be high-density integrated with excellent working ability even at such a high temperature. In addition, our unique but generic CQD microlaser fabrication method is very attractive and promising from a commercial standpoint where they can greatly reduce manufacturing cost and simplify the manufacturing process, thereby benefiting their large-scale industrial production. In other words, these highly efficient solution-preparation processes do not need complex processing techniques and expensive processing equipment, the costs are mainly the low-priced materials. This cost-effective manufacturability and the flexible integration capability pave a new route and promise great potential in the advancement of CQD microlasers from laboratory to industrialization. In addition, ever since the first demonstration of stimulated emission from CQDs, the pursuit of electrically pumped CQDs lasing has become the subject of intense research. Interestingly, our CQDAMs can serve as both gain medium and optical cavity, which can be readily incorporated into the electroluminescent architecture as an emitting layer to enable electrically pumped nanolasers. In fact, the realization of electro-induced microlaser is a great challenge, and more complex problems need to be solved, which is also an important content of our future research.

In summary, we achieved excellent lasing performance with high packing density, high coupling efficiency, high *Q* factor, and low-lasing threshold by fabricating close-packed CQD-assembled microspheres. The CQDAMs embedded in the silica matrix show amazing heat resistance, obtaining the stable single-mode microlaser at an operative temperature of up to 450 K. In addition, these CQD lasers, based on low-cost and mass-produced solution-processable methods, can be highly integrated into a micro-substrate, promising for economical high-temperature on-chip light sources.

## Materials and methods

### Sample fabrication and characterization

Colloidal CdSe/ZnS, CdSe/CdS, and ZnCdS/ZnS quantum dots are commercially available (Xingzi (Shanghai) New Material Technology Development Co., Ltd.). The assemble technique combining with the sol–gel solidification method is used to fabricate close-packed CQD-assembly optical microsphere cavities embedded in the silica matrix. First, tetraethylorthosilicate (TEOS, 2.6 mmol, 0.6 mL) and ethanol (5 mmol, 0.3 mL) were mixed under magnetic stirring in an atmospheric environment for 3 min. Then, the mixture was added to another beaker in which contained CQD (0.1 mmol, 0.2 mL) and 3-mercaptopropyltrimethoxysilane (3-MPS, 0.2 mmol, 0.2 mL), keeping the mixture on stirring for about 5 min. At the same time, a small amount (1 mmol, 0.1 mL) of hydrochloric acid was added to deliberately break the equilibrium of CQD in the sol process, make a rapid self-aggregation of CQD to form close-packed CQD clusters, and slowly evaporating the solution enables CQD of the similar size to aggregate more tightly, thus to achieve regular geometrical CQDAMs. The size of CQDAM can be controlled by varying the concentration of the CQD and the stirring rate. Next, propylamine acts as a basic catalyst to reduce the solidification time from few days to several minutes and neutralize the mixture to alkalescent to protect the CQDs. Furthermore, the long organic chains of 3-MPS tend to combine with the sulphion (S^2−^) on the CQDs surface shell, which protects the stability of the CQDs, as well as cross-link the CQDs with the covalent silica network to eliminate interface incompatibilities. The sol rapidly solidifies through a gel process to form a protective matrix and we obtained the CQD-assembly microspheres embedded in the silica matrix. Transmission electron microscopy (TEM and HAADF-TEM) measurements were performed on a Tecnai G2 F20 S-TWIN operated at 200 KV. All the samples are previously dropped on the clean bare wafer with fine concentration and later transfer onto a 200-mesh copper TEM grid by spot-cleaning.

### Experimental configures

The samples are put in a Dewar (77–475 K, Janis ST-500) with a temperature controller (cryocon 22C) for all optical experiments. The excitation source is a 400-nm femtosecond pulsed laser (~40 fs, 10 KHz). The PL signals (Figs. 2a, b, d, 3a, b, d, and 4d) are collected by a ×50 microscopy objective (NA = 0.50) in a confocal fluorescence detection system by the spectrometer (LabRAM HR Evolution). The time-resolved PL measurements (Fig. 2c) are obtained by a streak camera with a picosecond-order time resolution (Optronis, SC-10). The interference image (Fig. 3c) is measured by a set of self-built Michelson interferometers.

### Theoretical model

For semiconductor microlasers, the photon-stimulated amplification process can be simulated by rate equations,

1 $$\begin{array}{*{20}{c}} {\frac{{{\mathrm{d}}n}}{{{\mathrm{d}}t}} = - \Gamma _{{\mathrm{sp}}}n - \Gamma _{{\mathrm{sp}}}f\left( {n - n_{{\mathrm{pi}}}} \right)m - \Gamma _{{\mathrm{nr}}}n + {\mathrm{Pump}}\left( t \right)} \end{array}$$

2 $$\begin{array}{*{20}{c}} {\frac{{{\mathrm{d}}m}}{{{\mathrm{d}}t}} = \Gamma _{{\mathrm{sp}}}fn + \Gamma _{{\mathrm{sp}}}f\left( {n - n_{{\mathrm{pi}}}} \right)m - \Gamma _{\mathrm{c}}m} \end{array}$$

Here, *n* is the number of excitons, and Γ_sp_ is the spontaneous radiation rate. *m* is the number of emitted photons that are coupled into the optical WG mode, and *f* is the coupling efficiency. *n*_pi_ is the particle number corresponding to the population inversion density. Γ_nr_ is the nonradiative decay rate of the exciton reservoir. Γ_c_ is the decay rate of *m* leaking out of the cavity. Pump(*t*) is the initially pulsed excitation term with a Gaussian shape. Note that the stimulation term of Γ_sp_ *f*(*n*−*n*_pi_)*m* is valid only when the population inversion condition is satisfied, i.e., *n* ≥ *n*_pi_. The time-integrated PL intensity *I* can be expressed as:

3 $$I \propto {\int} {\left[ {{{\Gamma }}_{{\mathrm{sp}}}n(1 - f) + {{\Gamma }}_{\mathrm{c}}m} \right] \cdot {\mathrm{d}}t}$$

When the temperature *T* increases, the phonon-related scatterings of excitons and the shape distortion-induced decay of cavity photons are enhanced, while the oscillator strength of excitons decreases. They change the values of parameters of Γ_nr_, Γ_c_, Γ_sp_, respectively. Based on the above theoretical model, the parameters are designated as follows, Γ_sp_ = 0.095 ns^−1^, Γ_nr_ = 3.3 ns^−1^, Γ_c_ = 0.85 ps^−1^, *f* = 0.05, *n*_pi_ = 10^5^. By changing the initial excitation number, the power-dependent PL intensity in Fig. 2b could be well simulated. In addition, simplified relationships are used to fit the temperature-dependent data in Fig. 3b, i.e., Γ_sp_ = 0.095−2.3 × 10^−4^ × (*T* − 450) ns^−1^, Γ_nr_ = 3.3 + 5 × 10^−3^ × (*T* − 450) ns^−1^, and Γ_c_ = 2.05 + 3.9 × 10^−3^ × (*T* − 450) ps^−1^. By changing the temperature *T*, the temperature-dependent laser threshold in Fig. 3b could be calculated.

## Supplementary information

Supplementary Information for Ultrastable low-cost colloidal quantum dot microlasers of operative temperature up to 450 K

## Data Availability

All data in the paper and the supplementary materials are available.
