# Supplementary material for: Ultrastable low-cost colloidal quantum dot microlasers of operative temperature up to 450 K
Source: Light Sci Appl. 2021 Mar 18;10:60. doi: 10.1038/s41377-021-00508-7 (PMC7969957; doi:10.1038/s41377-021-00508-7)
Supplement: Supplementary file 1 — Supplementary Information for Ultrastable low-cost colloidal quantum dot microlasers of operative temperature up to 450 K [file 41377_2021_508_MOESM1_ESM.docx]

**Supplementary Information for**

**Ultrastable low-cost colloidal quantum dot microlasers of operative temperature up to 450 K**

Hao Chang^1,2,3,4+^, Yichi Zhong^1,2,3+^, Hongxing Dong^1,3,5*^, Zhenyu Wang^2^, Wei Xie^2*^, Anlian Pan^6^ and Long Zhang^1,3,5*^

*^1^ Key Laboratory of Materials for High-Power Laser, Shanghai Institute of Optics and Fine Mechanics, Chinese Academy of Sciences, Shanghai 201800, China.*

*^2^ State Key Laboratory of Precision Spectroscopy, School of Physics and Electronic Science, East China Normal University, 200241 Shanghai, China.*

*^3^ Hangzhou Institute for Advanced Study, University of Chinese Academy of Sciences, Hangzhou 310024, China.*

*^4^ Center of Materials Science and Optoelectronics Engineering, University of Chinese Academy of Sciences, Beijing 100049, China.*

*^5^ CAS Center for Excellence in Ultra-intense Laser Science, Shanghai 201800, China.*

*^6^ Key Laboratory for Micro-Nano Physics and Technology of Hunan Province, State Key Laboratory of Chemo/Biosensing and Chemometrics, College of Materials Science and Engineering, Hunan University, Changsha 410082, China.*

^+^These authors contributed equally to this work.

*Corresponding author. Emails: [hongxingd@siom.ac.cn](mailto:hongxingd@siom.ac.cn); [wxie@phy.ecnu.edu.cn](mailto:wxie@phy.ecnu.edu.cn); [lzhang@siom.ac.cn](mailto:lzhang@siom.ac.cn)

**Part I: Extended Experimental Data of sample characterization and room-temperature lasing.**

Fig. S1. Characteristics of CdSe/ZnS CQD.

Fig. S2. Characteristics of CQDAMs.

Fig. S3. Synthesis schematic of the CQDAMs embedded in silica matrix.

Fig. S4. Elemental analysis of CQDAMs.

Fig. S5. Single-mode lasing characteristics of a CQDAM at room temperature.

Table S1. The lasing performance of present typical CQD microlasers.

**Part Ⅱ: Discussions for the optimization of the intrinsic gain characteristics from CQD solution to CQDAM structure.**

Fig. S6. Temperature-dependent PL characteristics of the three different CQD structures.

Table S2. Fitting results of the temperature-dependent PL data.

**Part ⅡI: Introduction of the fitting functions and parameters.**

Fig. S7. The lasing spectra of three typical CQDAMs with different sizes.

**Part I: Extended Experimental Data of sample characterization and room-temperature lasing.**


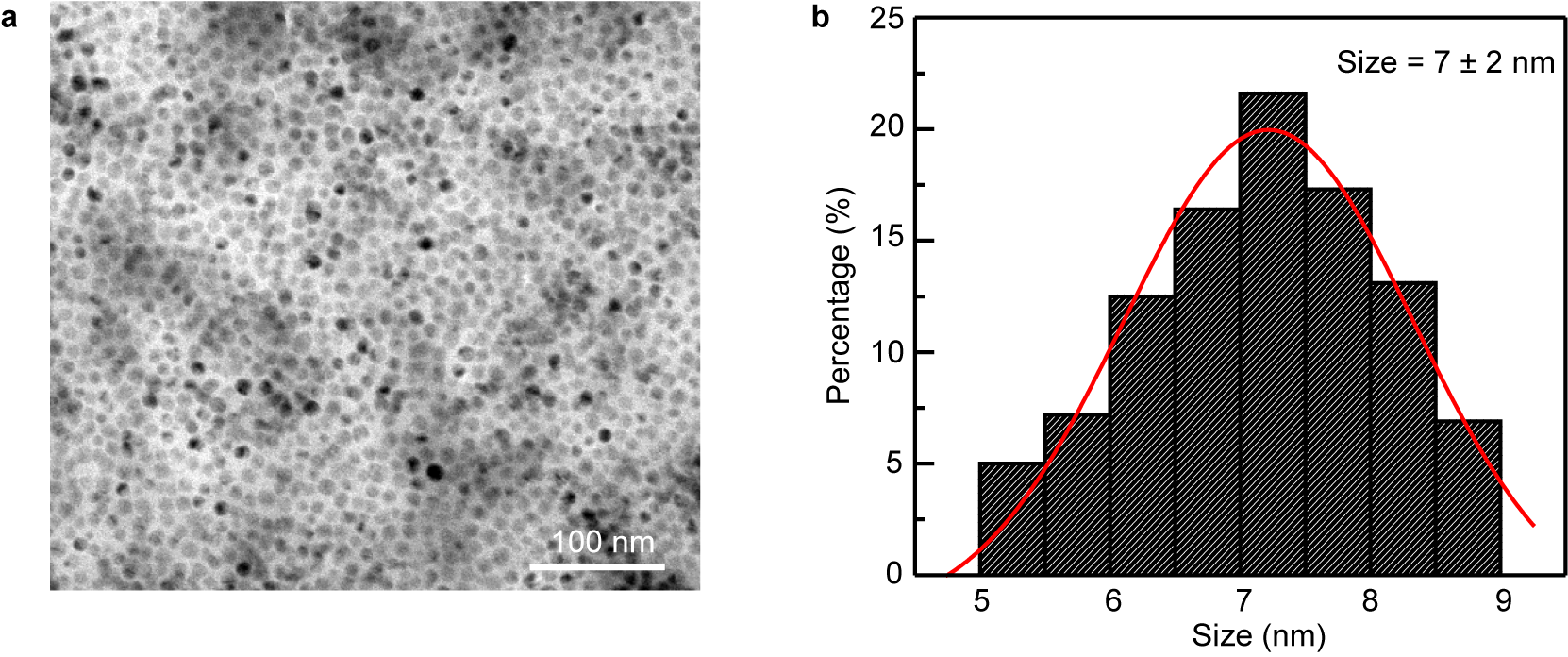


**Fig. S1. Characteristics of CdSe/ZnS CQD. a**, Low resolution TEM of dispersed CQD. **b**, Histogram of CQD size distribution from Fig. S1a. The average size is 7 nm, with deviation of ± 2 nm.


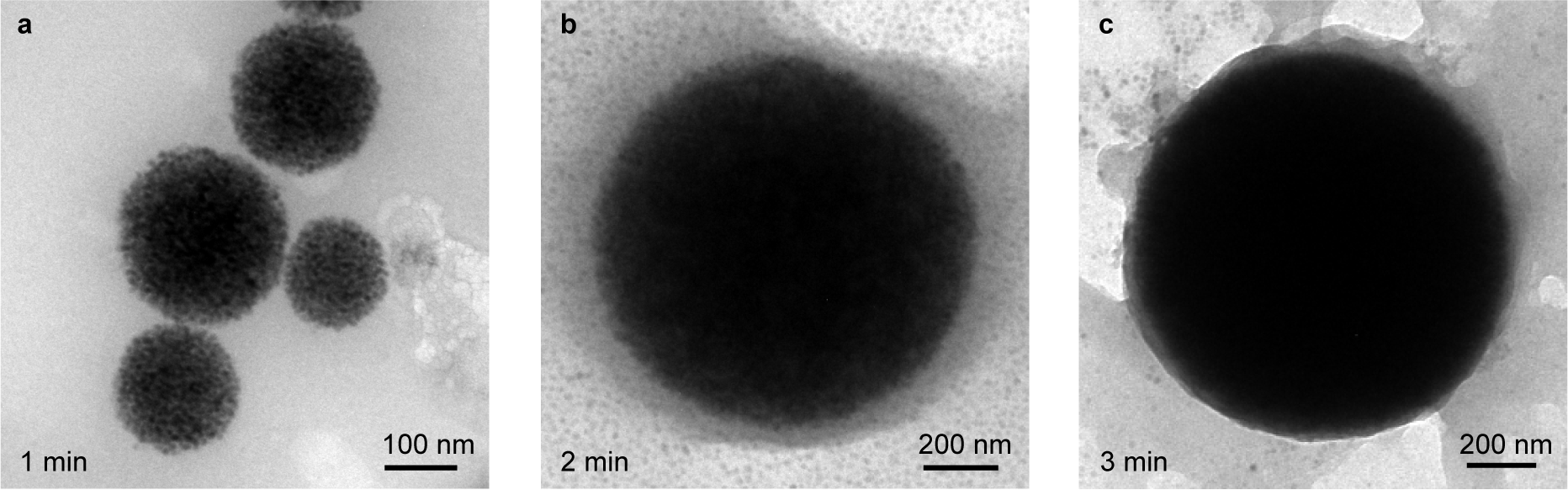


**Fig. S2. Characteristics of CQDAMs.** TEM images of aggregate evolution for different CQD structures varying with reaction times. Dispersed CQD aggregated to the CQD cluster (a,b) and finally to the close-packed CQD microsphere (c) with regular geometry.


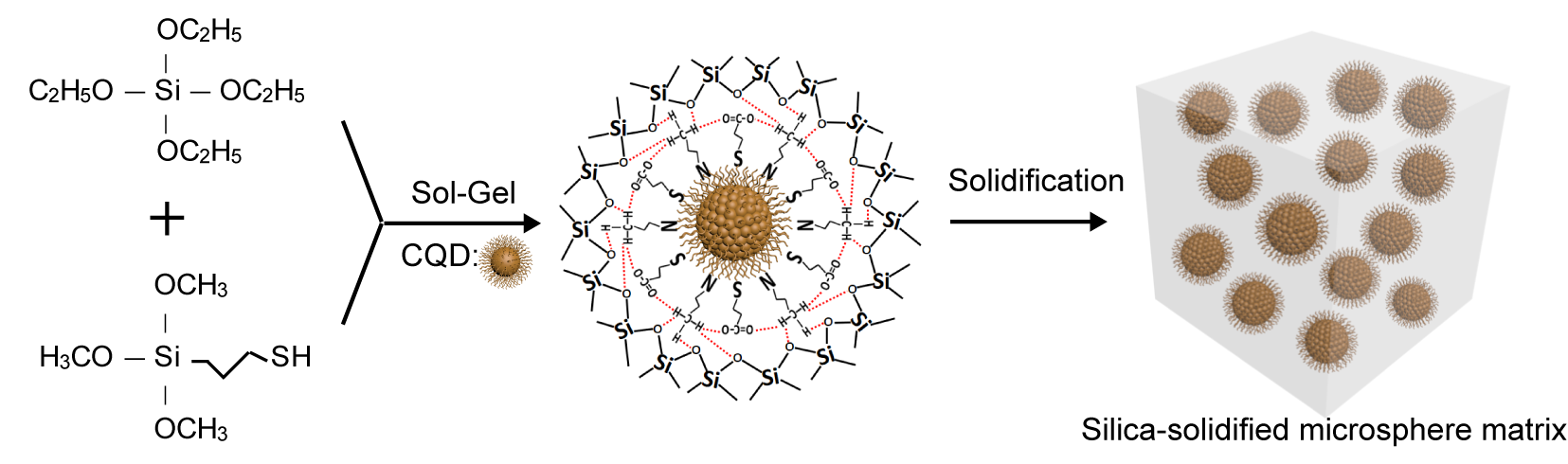


**Fig. S3. Synthesis schematic of the CQDAMs embedded in silica matrix.** Two silanization reagents tetraethylorthosilicate (TEOS) and 3-mercaptopropyltrimethoxysilane (3-MPS) are chosen as silica precursors to form the silica network. Dispersed CQD self-assembled to the CQDAMs and then solidified in the silica matrix in sol-gel process.

High thermal tolerance means the CQDAMs solidified in the silica matrix can exist stably and maintain good optical properties over a higher temperature range. The thermally induced interfacial tension and atomic dislocation is the most likely origin of nonradiative trapping centers and surface defects of CQDs. And, more intense molecular thermal motion at high temperature will lead the capping ligands of the CQDs detached, which also degraded the optical properties of CQDs^1-3^. When solidified CQDs samples in the silica-based network, the inactivation ligands cannot detach and leave the surface, thus forestalling the degradation of CQDs. The inorganic matrix also eliminates the surface defect states and thus ensures the excellent optical performance of CQDs samples at elevated temperatures^4,5^.


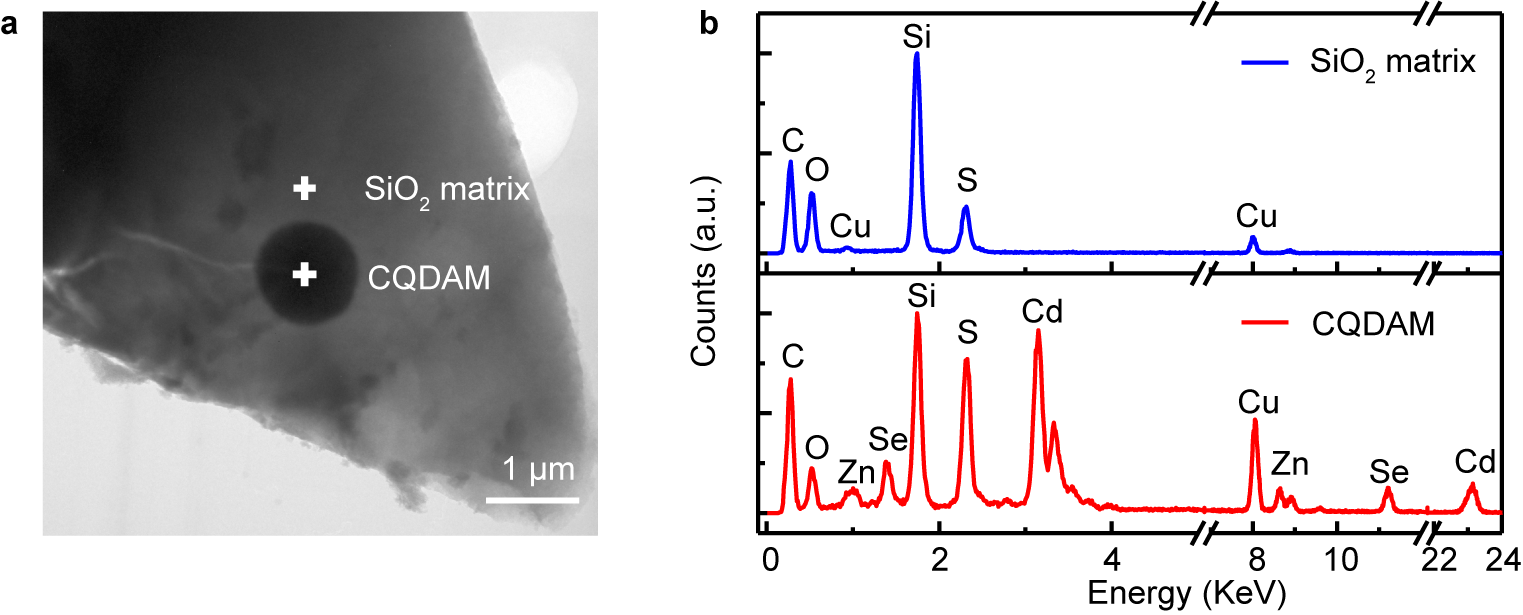


**Fig. S4. Elemental analysis of CQDAMs. a**, TEM image of an individual CQD microsphere embedded in silica matrix. **b**, TEM-EDX of the marked position in Fig. S4a, showing the different elemental compositions.


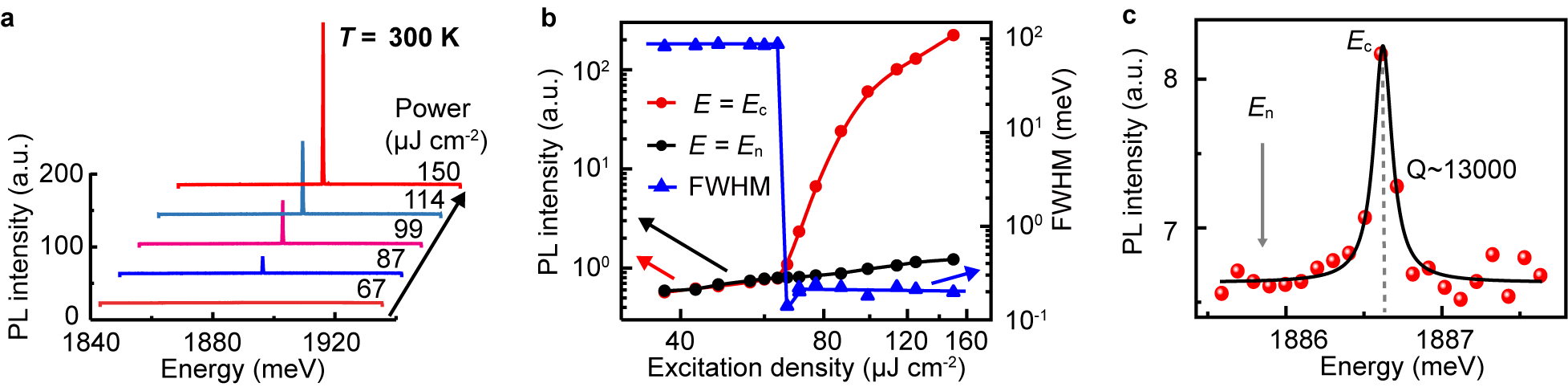


**Fig. S5. Single-mode lasing characteristics of a CQDAM at room temperature.** **a,** Pump intensity-dependent emission spectra of a typical CQDAM at room temperature. **b,** The excitation density dependence of PL intensity at the cavity resonance energy (red line) and the nonresonant energy (black line). Plots of emission FWHM of PL spectra vs excitation density (blue line). **c,** Lorentzian fitting of the lasing peak with a FWHM of ∼ 0.14 meV, corresponding to the Q factor of ∼ 13000.

PS: The Q factor of optical resonator is usually obtained from the transmission/reflection spectrum of the cavity. But in the particular case of our experiment, the resonant cavity itself is full of gain medium, i.e., the cavity is made by the gain medium. In this case, if the excitation power falls below the lasing threshold, the gain medium will have a strong absorption on the light fields with the wavelength range at the exciton energy level. We cannot find the cavity mode in the PL spectra below the lasing threshold because the photon lifetime in the resonant cavity is so short (< 50 fs, corresponding to the attenuation length smaller than 5 μm). The broadened PL peak below threshold originates from the linear emission of the gain medium, which is not related with the cavity mode. However, when the excitation power is close to the lasing threshold (at least, reaching the population inversion condition), the absorption of photons by the cavity/gain medium will be compensated. Above the threshold, the cavity photon lifetime is determined by the geometrical regularity of cavity and the smoothness of morphology surface. In this case, the Q-factor indicates the nature of optical confinement ability of the cavity structure, and the cavity absorption is negligible.

The abrupt linewidth narrowing of the PL peak at the lasing threshold point (**Fig. S5b**) is a complex behavior relating with the normal emission of the gain medium, the mode selection effect during the lasing process and the photon confinement ability of cavity structure. When the excitation power exceeds the lasing threshold, lasing action emerges at the cavity mode, as seen the new sharp peak in the PL spectrum. The FWHM of lasing peak is related with two parts, i.e., the intrinsic linewidth determined by the spontaneous radiation rate of excitons, and the modulated linewidth originating from the mode selection in the cavity system. Due to the small spontaneous radiation rate of excitons $\Gamma_{\mathrm{ex}}$ < 0.001 ps^-1^, the FWHM of lasing peak is mainly determined by the later. In our experiment, the FWHM of lasing peak narrows to about 0.6-1.3 meV, which suggests that after excluding the absorption effect of the gain medium, the lifetime of cavity photons $\tau_{c}$ is about 0.9-2 ps.

**Table S1. The lasing performance of present typical CQD microlasers.**

| Material | Resonator | Q factor | Threshold (μJ cm^-2^) | Temperature | Pump source | Ref. |
| --- | --- | --- | --- | --- | --- | --- |
| CdSe/Cd_x_Zn_1-x_S | Film | - | 4.4 | RT | 400 fs, 1 kHz | 6 |
| CdSe/CdS | Film | - | 29 | RT | 400 fs, 1 kHz | 7 |
| CdSe/CdS | Film | - | 2200 | RT | 532 ps, 2.5 kHz | 8 |
| CdSe/CdS/ZnS | Film | - | 891 | RT | 532 ps, 100 Hz | 9 |
| CdSe/CdS/ZnS | Film | - | 17100 | RT | 1300 fs, 1 kHz | 10 |
| CsPbBr_3_ | Film | - | 1.2 | RT | 400 fs, 1 kHz | 11 |
| CsPbBr_3_ | Film | 520 | 40 | RT | 400 fs, 1 kHz | 12 |
| CsPbBr_3_ | Film | - | 143 | RT | 400 fs, 1 kHz | 13 |
| CsPbBr_3_ | Film | - | 192 | RT | 400 fs, 1 kHz | 14 |
| CsPbX_3_ | Film | - | 970 | RT | 400 fs, 1 kHz | 15 |
| CsPbBr_3_ | Film | - | 2800 | RT | 800 fs, 1 kHz | 16 |
| CdZnS/ZnS | WGM | - | 25200 | RT | 395 ns, 20 Hz | 3 |
| CdZnS/ZnS | WGM | 2500 | 10.8 | RT | 400 ns, 20 Hz | 17 |
| CsPbBr_3_ | WGM | 1637 | 11.6 | RT | 400 fs, 1 kHz | 18 |
| CsPbBr_3_ | WGM | - | 22 | RT | 400 fs, 1 kHz | 19 |
| CsPbBr_3_ | WGM | 920 | 200 | RT | 450 ns, 10 Hz | 20 |
| CsPbBr_3_ | WGM | 1060 | 800 | RT | 800 fs, 1 kHz | 21 |
| CsPbBr_3_ | DBRs | 580 | 0.39 | RT | 400 fs, 1 kHz | 22 |
| CsPbX_3_ | DBRs | 920 | 11 | RT | 400 fs, 1 kHz | 23 |
| CsPbBr_3_ | DBRs | - | 2000 | 370 K | 800 fs, 1 kHz | 24 |
| cg-QDs | DFB | - | 5.5 | RT | 400 fs, 1 kHz | 25 |
| CdSe/CdZnS/ZnS | DFB | - | 83 | RT | 400 fs, 1 kHz | 26 |
| CdSe/ZnCdS | DFB | - | 60 | RT | 400 fs, 100 kHz | 27 |
| CsSnI_3_ | DFB | 3000 | 800 | RT | 532 ns, 10 Hz | 28 |
| Ⅱ-Ⅵ CQDs | WGM | 3500-13000 | 60-190 | RT-450 K | 400 fs, 10 kHz | Our work |

The lasing performance of present typical CQD microlasers are listed in **Table S1**. Compared to the reported CQD microlasers, single-mode lasing in CQDAMs exhibit high Q factor and relatively low lasing threshold, which maintains excellent lasing performance even at 450 K. Generally, the Q factor of a traditional dielectric microcavity decreases drastically due to the greater radiation loss in a smaller cavity, which also leads to a higher lasing threshold^29,30^. In our work, the good lasing performance of such small CQDAMs may explain as follows. Firstly, the self-assembled CQDs almost reach the high limit of packing density, ensuring sufficient optical gain. Secondly, such CQDAM samples are used both as gain materials and as optical microcavities, fully improving the light-matter coupling efficiency. Finally, the spherical WGM microcavity can effectively improve the confinement ability of cavity photons.

**Part Ⅱ: Discussions for the optimization of the intrinsic gain characteristics from CQD solution to CQDAM structure.**

Our original idea of sample fabrication from dispersed CQD to CQDAMs is efficient for the optimization of the intrinsic physical parameters of the gain medium to overcome the PL quenching effect at high temperature. **Fig. S6a** shows contour plots of the temperature-dependent PL spectra from the CQD solution (top), CQD cluster (middle), and CQDAM (down). In each structure, the emission peak is redshifted, the PL intensity decreases, and the spectra become broader with temperatures ranging from 77 K to 475 K. Interestingly, the CQDAMs have the smallest redshift energy of the PL peak (**Fig. S6b**), the smallest increase of peak FWHM (**Fig. S6c**), and the smallest reduction of PL intensity (**Fig. S6d**) compared to those of other structures, which provide favorable conditions for achieving a stable laser at high temperature.


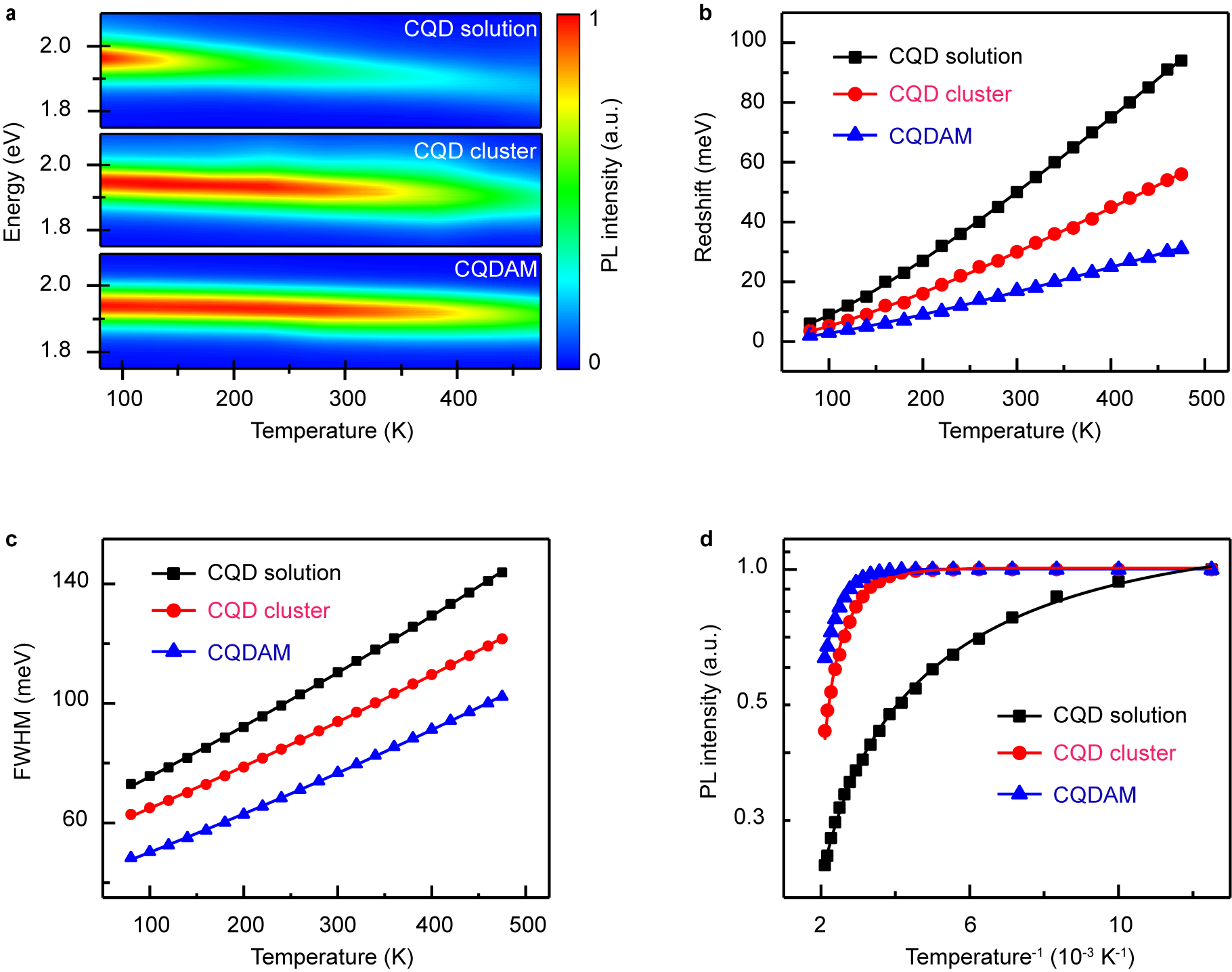


**Fig. S6. Temperature-dependent PL characteristics of the three different CQD structures. a,** Contour plots of the temperature-dependent emission from different CQD structures with the temperature varied from 77 K to 475 K under the same excitation conditions. **b-d,** Evolution of emission peak redshifts (b), spectral full-width at half-maximum (FWHM) (c) and PL intensity (d) for the three different CQD structures with the increase of temperature.

The temperature-dependent redshift of the PL peak originates from the thermal lattice expansion, which leads to a shrinkage of the energy bandgap. We obtained the smallest temperature coefficient (*α*) of 0.1 meV K^-1^ for CQDAMs from the experimental data (**Table S2**). The smaller *α* indicates that the effect of temperature on the bandgap fluctuation is weaker, suggesting a better thermal stability of CQDAMs in the silica matrix at elevated temperature environments. The peak FWHM is mainly related to the inhomogeneous sizes of the CQD and the scattering between the excitons and optical/acoustic phonons. The FWHM at low temperature is mainly determined by the former. The PL spectrum of CQDAM has the smallest FWHM of 77 meV compared with the 110 meV for CQD solution and 94 meV for CQD cluster, suggesting a smallest deviation in the size distribution of the assembled CQD. At high temperatures, the FWHM is significantly influenced by the nonradiative scattering rate between excitons and thermally induced phonons, which decreases the nonradiative lifetime and the radiative efficiency of excitons. This is one of the reasons for the quenching of the PL intensity. Meanwhile, the defect states and other nonradiative centers also make notable contributions. The PL quenching effect is a critical issue that hinders the high-temperature applications of CQD. For solidified CQDAMs, additional surface passivation eliminates surface defects, and the closely packed microsphere structure significantly decreases the density of inner defects, which significantly overcome the PL quenching effect and ensure strong optical gain for the high-temperature lasing.

**Fig. S6b** shows the monotonic redshifts of emission peak energies with increasing temperature for the three CQD structures owing to the thermal lattice expansion and increased electron-phonon interaction. The solid lines in **Fig. S6b** are the least-squares simulations to the experimental data using the Varshni equation (**Eq. (1)**), which describes the temperature dependence of the semiconductor bandgaps.

$$\begin{aligned} \Delta E=E_{g}\left( 0 \right)-E_{g}\left( T \right)=\alpha\frac{T^{2}}{\beta+T}\#(1) \end{aligned}$$

The values of the extracted parameters are summarized in **Table S2**, where *E*_g_(0) is the band gap at 0 K, *α* is the temperature coefficient, and *β* approximates the Debye temperature of the material. The temperature coefficient *α* is strongly dependent on and is inversely proportional to the size of the CQD, the smaller *α* means the effect of temperature on the bandgap fluctuation is weaker, suggesting a better thermal stability of CQDAM in silica matrix at elevated temperature environments. The *β* values with little difference, which corresponds to some average phonon energy, might indicate the increased coupling of the optical transition to lower energy acoustic phonons and the reduced coupling to longitudinal optical (LO) phonons in different CQD structures.

**Fig. S6c** shows the FWHM of lasing peaks as the temperature increases. The FWHM is closely related with the inhomogeneity of CQD size and the scatterings between excitons and optical/acoustic phonons. The experimental data of PL broadening are well simulated by **Eq. (2)**,

$$\begin{aligned} \Gamma\left( T \right)=\Gamma_{\mathrm{inh}}+\Gamma_{\mathrm{AC}}T+\Gamma_{\mathrm{LO}}\left( e^{{E_{\mathrm{LO}}}/{k_{B}T}}-1 \right)^{-1}\#(2) \end{aligned}$$

where *Г*_inh_ represents the temperature-independent intrinsic inhomogeneous linewidth. *Г*_AC_ is the coefficient of the acoustic phonon-exciton interaction, *Г*_LO_ is the LO phonon-exciton coupling coefficient, and *E*_LO_ is the LO-phonon energy. The emission linewidth broadening originates from two contributions: the temperature-independent inhomogeneous broadening due to the dispersion in size, shape, elemental composition, etc., and the temperature-dependent homogeneous broadening due to acoustic and LO phonon-exciton interactions. The smaller value of *Г*_inh_ represents the uniformly size distribution of the nanocrystals, indicating the size selection in the formation of CQDAMs.

As shown in **Fig. S6d**, the temperature-dependent PL intensity of the three structures dropping from 100.0% to 21%, 40%, and 58% at 475 K relative to its original PL intensity at 77 K. These results can be fitted by the Arrhenius equation:

$$\begin{aligned} I\left( T \right)=\frac{I\left( 0 \right)}{1+Ae^{{-E_{b}}/{k_{B}T}}}\#(3) \end{aligned}$$

where *I*(0) is the intensity at low temperature, *E*_b_ is the effective exciton binding energy, *k*_B_ is Boltzmann constant and *A* is a constant. *E*_b_ is an intrinsic physical parameter for semiconductor. If the exciton binding energy is smaller than the thermal energy *E*_k_ at a specific temperature *T* (*E*_k_ ∼ *k*_B_*T*), the exciton is dissociated to individual electron and hole, otherwise the exciton is stable. Here, the physical parameters of *E*_b_ and *E*_k_ can be analogous with the radiative rate $\Gamma_{\mathrm{sp}}$ and nonradiative rate $\Gamma_{\mathrm{nr}}$ in the theoretical model in Methods. The substantial differences can be seen in the degree of PL quenching at elevated temperature. The CQDAMs in silica matrix have the largest *E*_b_ value than other structures owing to the additional surface passivation eliminates the surface defects, which ensures strong optical gain and provides powerful conditions for stimulated radiation.

Regarding the shift trend of the lasing peak in **Fig.3a**, we discussed as follows. In general, the resonant modes of a spherical WGM microcavity can be simply expressed as:

$$\begin{aligned} {\pi D}/\left( \lambda/N \right)=\nu\#(4) \end{aligned}$$

where *D* denotes the diameter of CQDAM; *λ* is the wavelength of the resonant peak; *ν* and *N* are the mode orders and the refractive index of the medium, respectively. In our experiment, the size of the CQDAM varies very little and only one lasing mode survives in the gain region, so the mode order ν is unchanged. Besides, the refractive index (*N*) and energy gap (*E*_g_) are related as follows according to the Moss relation:

$$\begin{aligned} N^{4}E_{g}=\mathrm{constant}\#(5) \end{aligned}$$

In our experiment, the PL emission peak energy of CQDAM monotonic redshifts with increasing temperature, corresponding to the energy gap (*E*_g_) of 1938 meV (80 K) and 1910 meV (450 K), and thus, the refractive index (*N*) also changes as the temperature increases, leading to the shifts of the resonant peak. As the lasing emission peak energy is 1865.38 meV at 80 K in our experiment, calculated with the **Eq. (4)** and **Eq. (5)**, the theoretical value of lasing emission peak energy at 450 K should be 1858.61 meV, which is consistent with our experimental result *E*_lasing_(450 K) = 1858.73 meV.

**Table S2. Fitting results of the temperature-dependent PL data.**

| Sample  Parameters | | CQD solution | CQD cluster | CQDAM |
| --- | --- | --- | --- | --- |
| Eq. (1) | *α* (10^-4^ eV K^-1^) | 3.12 ± 0.16 | 1.81 ± 0.09 | 1.06 ± 0.05 |
|  | *β* (K) | 200 ± 10 | 230 ± 10 | 240 ± 15 |
| Eq. (2) | *Г*_inh_ (meV) | 66.8 ± 3.3 | 57.4 ± 2.8 | 43.2 ± 2.1 |
|  | *Г*_AC_ (μeV K^-1^) | 74.6 ± 3.7 | 62.5 ± 3.1 | 56.2 ± 2.8 |
|  | *Г*_LO_ (meV) | 34.2 ± 1.7 | 28.4 ± 1.4 | 26.2 ± 1.3 |
|  | *E*_LO_ (meV) | 24.8 ± 1.2 | 25.6 ± 1.2 | 25.2 ± 1.2 |
| Eq. (3) | *E*_b_ (meV) | 38 ± 2 | 182 ± 10 | 220 ± 10 |

**Part ⅡI: Introduction of the fitting functions and parameters.**

**(1) Fitting parameters of Fig. 2c**

We fit the two data curves (0.8 *P*_th_, and 1.5 *P*_th_) shown in **Fig. 2c** by using **Eq. (6)**. The decay time and the proportion of fast (*τ*_1_) and slow (*τ*_2_) decay components are obtained. When the excitation density increases above *P*_th_, a rapid carrier dissipation is observed due to the stimulated radiation behavior.

$$\begin{aligned} y=A_{1}e^{\frac{-x}{\tau_{1}}}+A_{2}e^{\frac{-x}{\tau_{2}}}\#(6) \end{aligned}$$

| Excitation density | *τ*_1_ (ps) | $\frac{A_{1}}{{(A}_{1}+A_{2})}$(%) | *τ*_2_ (ps) | $\frac{A_{1}}{{(A}_{1}+A_{2})}$(%) |
| --- | --- | --- | --- | --- |
| 0.8 *P*_th_ | 134 | 65.3 | 1946 | 34.7 |
| 1.5 *P*_th_ | 12.3 | 97.8 | 288.0 | 2.2 |

**(2) Numerical simulations of the inset in Fig. 2a or the data in Fig. 2d.**

Two/three-dimensional (2/3D) electric field distribution is simulated by COMSOL Multiphysics. We use the module of wave optics to calculate the electric field distribution of characteristic frequency. The resonant frequency of the cavity can form the stable standing wave field distributions, with high Q factor. For the inset in **Fig. 2a**, the 3D model is a microsphere (Radius: *R*_1_ = *R*_2_ = 0.9*R*_3_ = 590 nm) wrapped in a cube with a radius of 10 μm, and the outermost part is perfect absorption layer. The refractive index $n$ of materials from the microsphere to the outsides are 2.5, 1.5 and 1.0, respectively. The high-Q WG mode is confined in the circular plane constructed by *R*_1_ and *R*_2_.

For the simulation results in **Fig. 2d**, a simplified 2D model is used, i.e., an adjustable size circle (*n* = 2.5) wrapped in a square (*n* = 1.5, *R* = 10 μm). The relationship between sample size and mode space can be simulated. More details are shown as follows,


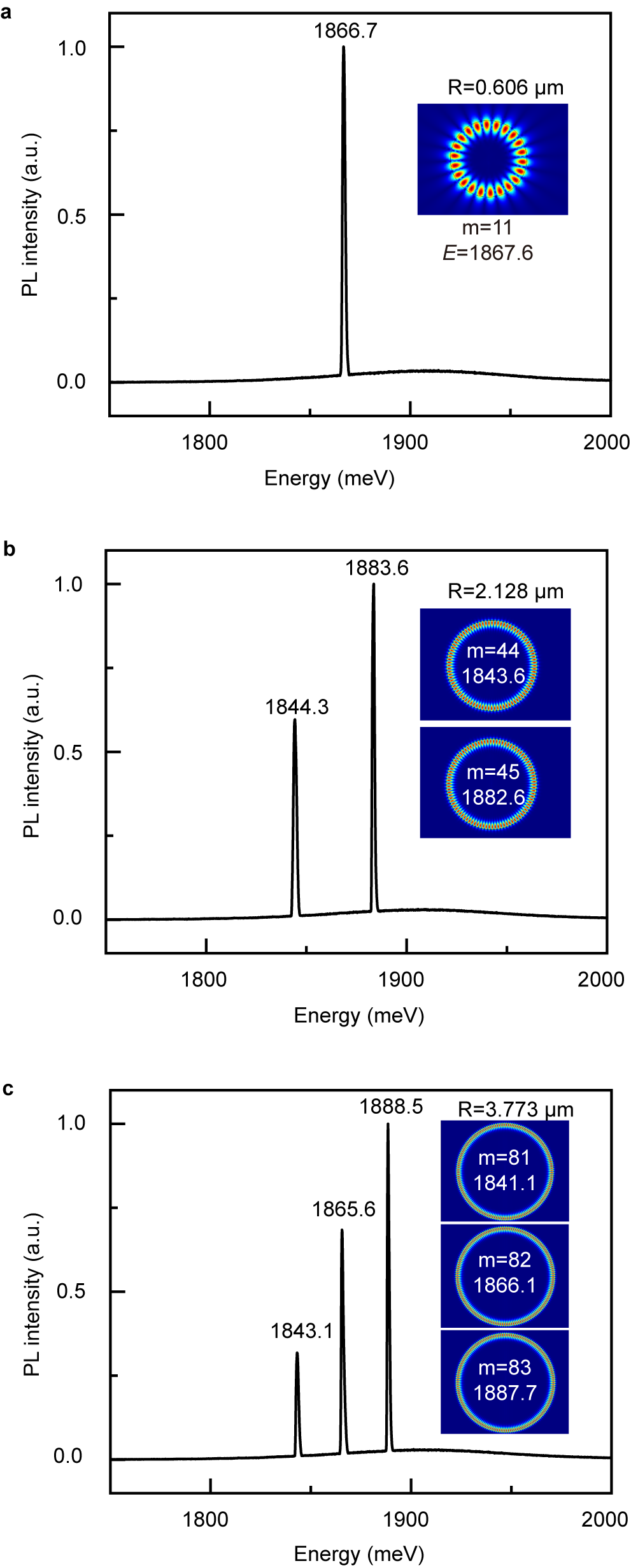


**Fig. S7. The lasing spectra of three typical CQDAMs with different sizes.** Insets: the theoretical field distribution of the resonant lasing modes by COMSOL simulation. Standing waves are confined in the microsphere optical cavity.

**(3) Fitting parameters of Fig. 3c for the interference fringe visibilities and the temperature-dependent coherence times.**

Coherent emission is an important feature of lasers. The fringe visibility data are fitted with Gaussian line shape (**Eq. 7**). The value of $\frac{A}{w\sqrt{0.5\pi}}$ can reflect the coherent components, which are 0.61, 0.37, 0.35 at 300 K, 370 K, 400 K, respectively. The optical signals also contain incoherent PL from spontaneous emission and the proportion of spontaneous emission is more at higher temperatures. The value of $\frac{w}{2}$ presents the coherent time, which are 5.38 ps, 0.85 ps, 0.44 ps at 300 K, 370 K, 400 K, respectively. The higher the temperature, the shorter of the coherent time.

$$\begin{aligned} y=y_{0}+\frac{A}{w\sqrt{\frac{\pi}{2}}}e^{\frac{{-2(x-x_{c})}^{2}}{w^{2}}}\#(7) \end{aligned}$$

| Operating temperature (K) | *y*_0_ | *A* | *x*_c_ (ps) | *w* (ps) |
| --- | --- | --- | --- | --- |
| 300 | -0.02 | 8.52 | -0.61 | 10.76 |
| 370  400 | -0.02  0.00 | 0.81  0.37 | 0.13  0.07 | 1.70  0.88 |

**References**

1 Zhao, Y. M. *et al*. High-temperature luminescence quenching of colloidal quantum dots. *ACS Nano* **6**, 9058-9067, doi:10.1021/nn303217q (2012).

2 Rowland, C. E. *et al*. Thermal stability of colloidal InP nanocrystals: small inorganic ligands boost high-temperature photoluminescence. *ACS Nano* **8**, 977-985, doi:10.1021/nn405811p (2014).

3 Wang, Y. *et al*. Blue liquid lasers from solution of CdZnS/ZnS ternary alloy quantum dots with quasi-continuous pumping. *Advanced Materials* **27**, 169-175, doi:10.1002/adma.201403237 (2015).

4 Li, J. Z. *et al*. Colloidal quantum-dot-based silica gel glass: two-photon absorption, emission, and quenching mechanism. *Nanoscale* **8**, 16440-16448, doi:10.1039/c6nr03268k (2016).

5 Jun, S., Lee, J. & Jang, E. Highly luminescent and photostable quantum dot-silica monolith and its application to light-emitting diodes. *ACS Nano* **7**, 1472-1477, doi:10.1021/nn3052428 (2013).

6 Lim, J., Park, Y. S. & Klimov, V. I. Optical gain in colloidal quantum dots achieved with direct-current electrical pumping. *Nature Materials* **17**, 42-49, doi:10.1038/nmat5011 (2018).

7 Lafalce, E. *et al*. Robust lasing modes in coupled colloidal quantum dot microdisk pairs using a non-Hermitian exceptional point. *Nature Communications* **10**, 561, doi:10.1038/s41467-019-08432-6 (2019).

8 Gollner, C. *et al*. Random Lasing with Systematic Threshold Behavior in Films of CdSe/CdS Core/Thick-Shell Colloidal Quantum Dots. *Acs Nano* **9**, 9792-9801, doi:10.1021/acsnano.5b02739 (2015).

9 Yu, J. H. *et al*. Electrically control amplified spontaneous emission in colloidal quantum dots. *Science Advances* **5**, eaav3140, doi:10.1126/sciadv.aav3140 (2019).

10 Wang, Y. *et al*. Stimulated emission and lasing from CdSe/CdS/ZnS core-multi-shell quantum dots by simultaneous three-photon absorption. *Advanced Materials* **26**, 2954-2961, doi:10.1002/adma.201305125 (2014).

11 Wang, Y. *et al*. Stable, Ultralow Threshold Amplified Spontaneous Emission from CsPbBr3 Nanoparticles Exhibiting Trion Gain. *Nano Letters* **18**, 4976-4984, doi:10.1021/acs.nanolett.8b01817 (2018).

12 Li, X., Wang, Y., Sun, H. & Zeng, H. Amino-Mediated Anchoring Perovskite Quantum Dots for Stable and Low-Threshold Random Lasing. *Advanced Materials* **29**, doi:10.1002/adma.201701185 (2017).

13 Hu, Z. P. *et al*. Enhanced Two-Photon-Pumped Emission from In Situ Synthesized Nonblinking CsPbBr3/SiO2 Nanocrystals with Excellent Stability. *Advanced Optical Materials* **6**, doi:10.1002/adom.201700997 (2018).

14 Pan, J. *et al*. Air-Stable Surface-Passivated Perovskite Quantum Dots for Ultra-Robust, Single- and Two-Photon-Induced Amplified Spontaneous Emission. *The* *Journal of Physical Chemistry Letters* **6**, 5027-5033, doi:10.1021/acs.jpclett.5b02460 (2015).

15 Tang, X. S. *et al*. Room temperature single-photon emission and lasing for all-inorganic colloidal perovskite quantum dots. *Nano Energy* **28**, 462-468, doi:10.1016/j.nanoen.2016.08.062 (2016).

16 Wang, Y. *et al*. Nonlinear Absorption and Low-Threshold Multiphoton Pumped Stimulated Emission from All-Inorganic Perovskite Nanocrystals. *Nano Letters* **16**, 448-453, doi:10.1021/acs.nanolett.5b04110 (2016).

17 Wang, Y. *et al*. Robust Whispering-Gallery-Mode Microbubble Lasers from Colloidal Quantum Dots. *Nano Letters* **17**, 2640-2646, doi:10.1021/acs.nanolett.7b00447 (2017).

18 Wang, Y. *et al*. All-Inorganic Colloidal Perovskite Quantum Dots: A New Class of Lasing Materials with Favorable Characteristics. *Advanced Materials* **27**, 7101-7108, doi:10.1002/adma.201503573 (2015).

19 Yakunin, S. *et al*. Low-threshold amplified spontaneous emission and lasing from colloidal nanocrystals of caesium lead halide perovskites. *Nature Communications* **6**, 8056, doi:10.1038/ncomms9056 (2015).

20 Lin, C. H. *et al*. Large-Area Lasing and Multicolor Perovskite Quantum Dot Patterns. *Advanced Optical Materials* **6**, doi:10.1002/adom.201800474 (2018).

21 Xu, Y. Q. *et al*. Two-Photon-Pumped Perovskite Semiconductor Nanocrystal Lasers. *Journal of the American Chemical Society* **138**, 3761-3768, doi:10.1021/jacs.5b12662 (2016).

22 Huang, C.-Y. *et al*. CsPbBr3 Perovskite Quantum Dot Vertical Cavity Lasers with Low Threshold and High Stability. *ACS Photonics* **4**, 2281-2289, doi:10.1021/acsphotonics.7b00520 (2017).

23 Wang, Y. *et al*. Solution-Processed Low Threshold Vertical Cavity Surface Emitting Lasers from All-Inorganic Perovskite Nanocrystals. *Advanced Functional Materials* **27**, doi:10.1002/adfm.201605088 (2017).

24 Wang, Y. *et al*. Solution-Grown CsPbBr3 /Cs4 PbBr6 Perovskite Nanocomposites: Toward Temperature-Insensitive Optical Gain. *Small* **13**, doi:10.1002/smll.201701587 (2017).

25 Roh, J. *et al*. Optically pumped colloidal-quantum-dot lasing in LED-like devices with an integrated optical cavity. *Nature Communications* **11**, 271, doi:10.1038/s41467-019-14014-3 (2020).

26 Todescato, F. *et al*. Soft-Lithographed Up-Converted Distributed Feedback Visible Lasers Based on CdSe-CdZnS-ZnS Quantum Dots. *Advanced Functional Materials* **22**, 337-344, doi:10.1002/adfm.201101684 (2012).

27 Dang, C. *et al*. Red, green and blue lasing enabled by single-exciton gain in colloidal quantum dot films. *Nature Nanotechnology* **7**, 335-339, doi:10.1038/nnano.2012.61 (2012).

28 Chen, L. J. *et al*. Wavelength-Tunable and Highly Stable Perovskite-Quantum-Dot-Doped Lasers with Liquid Crystal Lasing Cavities. *ACS Applied Materials & Interfaces* **10**, 33307-33315, doi:10.1021/acsami.8b08474 (2018).

29 Gargas, D. J. *et al*. Whispering gallery mode lasing from zinc oxide hexagonal nanodisks. *ACS Nano* **4**, 3270-3276, doi:10.1021/nn9018174 (2010).

30 Li, J. T. *et al*. Single Mode ZnO Whispering-Gallery Submicron Cavity and Graphene Improved Lasing Performance. *ACS Nano* **9**, 6794-6800, doi:10.1021/acsnano.5b01319 (2015).
